# SIN3 is critical for stress resistance and modulates adult lifespan

**DOI:** 10.18632/aging.100684

**Published:** 2014-08-07

**Authors:** Valerie L. Barnes, Abhineeth Bhat, Archana Unnikrishnan, Ahmad R. Heydari, Robert Arking, Lori A. Pile

**Affiliations:** ^1^Department of Biological Sciences, Wayne State University, Detroit, Michigan, 48202, USA; ^2^Department of Nutrition and Food Science Wayne State University, Detroit, Michigan, 48202,USA

**Keywords:** Drosophila, longevity, oxidative stress, SIN3, deacetylation

## Abstract

Coordinate control of gene activity is critical for fitness and longevity of an organism. The SIN3 histone deacetylase (HDAC) complex functions as a transcriptional repressor of many genes. SIN3-regulated genes include those that encode proteins affecting multiple aspects of mitochondrial function, such as energy production and stress responsiveness, important for health maintenance. Here we used *Drosophila melanogaster* as a model organism to examine the role of SIN3 in the regulation of fitness and longevity. Adult flies with RNA interference (RNAi) induced knockdown expression of *Sin3A* have reduced climbing ability; an activity that likely requires fully functional mitochondria. Additionally, compared to wild type, adult *Sin3A* knockdown flies were more sensitive to oxidative stress. Interestingly, media supplementation with the antioxidant glutathione largely restored fly tolerance to oxidative stress. Although *Sin3A* knockdown flies exhibited decreased longevity compared to wild type, no significant changes in expression of many well-categorized aging genes were observed. We found, however, that *Sin3A* knockdown corresponded to a significant reduction in expression of genes encoding proteins involved in the de novo synthesis of glutathione. Taken together, the data support a model whereby SIN3 regulates a gene expression program required for proper mitochondrial function and effective stress response during adulthood.

## INTRODUCTION

Eukaryotic DNA is packaged into chromatin of which the repeating unit is the nucleosome comprised of histone proteins wrapped by the DNA. Regulation of gene expression is impacted by the activity of histone modifying enzymes [1]. Histone acetyltransferases (KATs) and deacetylases (HDACs) directly modulate histone acetylation levels, which in turn affect gene activity. Histone lysine acetylation has long been correlated with transcription activation and the reverse, a deacetylated histone lysine residue, with transcription repression [2]. KAT and HDAC enzymes typically associate with other proteins to form stable complexes [3, 4]. The other factors often serve as a scaffold for complex assembly and/or facilitate the targeting of the associated histone modifying enzyme and activity to specific sites along the genome. SIN3 serves a scaffold function for assembly of the SIN3 complex, which contains the HDAC RPD3 [3]. SIN3 was first identified in yeast as a general regulator of transcription [5]. Subsequent work in yeast and metazoans demonstrated that the primary role of SIN3 is to repress transcription, though examples of involvement in activation have been reported [6-11].

Expression profiles of *Drosophila* and mouse cells having reduced SIN3 levels revealed that several nuclear encoded mitochondrial genes are subject to SIN3 regulation [8, 9]. Likely as a consequence of the altered expression of genes encoding proteins that function in the mitochondria, *Sin3A* knockdown in *Drosophila* S2 cells results in various aberrations linked to mitochondrial activity, including perturbed ATP production and respiration rates [12]. *Saccharomyces cerevisiae* Sin3 is dispensable for growth in fermentable media, however, when *sin3* mutants are cultured under conditions that require oxidative respiration, the cells grow very poorly [12]. Similar to the findings in *Drosophila* cells, ATP production and respiration are disrupted in yeast *sin3* mutants [12]. Taken together, data from multiple investigators using different model systems have found that SIN3 is necessary for regulation of key metabolic processes.

*Sin3A* is an essential gene in metazoans. Mice encode two *Sin3* genes, *mSin3a* and *mSin3b*. Genetic knockout of either of the *mSin3* genes results in embryonic lethality [8, 13, 14]. Data obtained through analysis of both null alleles and conditional RNA interference (RNAi) knockdown in *Drosophila* indicate that *Sin3A* is required for multiple stages of development including embryogenesis and larvagenesis [15-17]. Conditional knockdown in wing imaginal disc tissue results in a small and curved adult wing, indicating that SIN3 regulates key developmental pathways [18]. RNAi screens performed in cultured cells revealed that SIN3 is a putative regulator of neural and cardiac development as well as an active player in ERK and JNK signaling [19, 20]. A recent genetic screen to identify suppressors of the *Sin3A* knockdown curved wing phenotype found that genes involved in multiple processes during development, including regulation of the cell cycle, Wnt signaling, metabolism and transcription, interacted with *Sin3A* [21].

SIN3 is required for viability in *Drosophila* and mouse, possibly due to the fact that it is critical for cell cycle progression in both *Drosophila* and mammalian cells. Knockdown of *Sin3A* in *Drosophila* cultured cells by RNAi results in a G2 phase delay in cell cycle progression [22]. Additionally, microarray analysis of wild type and *Sin3A* knockdown cells identified differences in the expression of several cell cycle control genes [9]. Analysis of *Drosophila Sin3A* conditional mutants indicated that reduction of SIN3 during embryogenesis leads to death consistent with defects in cell cycle progression as opposed to disruption of specific developmental pathways [17]. Additionally, the curved wing phenotype associated with RNAi induced *Sin3A* knockdown is suppressed by overexpression of the G2/M regulator String, indicative of a cell cycle function [18]. Work with mammalian cells shows that loss of mSin3A causes changes in both G_1_ and G_2_/M phases of the cell cycle [13, 23]. The mammalian Sin3B complex is recruited to cell cycle genes in quiescent and early G1 cells [24, 25], and this activity is essential for cell cycle withdrawal [14]. Furthermore, mSin3 has been identified as a critical component in the permanent repression of cell cycle genes during differentiation [26]. Although not essential for yeast viability in normal conditions, ySin3 was shown to be important for accurate timing of Swi4/Swi6-regulated gene transcription during G1 and for activation of S phase in daughter cells [27]. Additionally, *S. cerevisiae* *SIN3* was found to be important for cell cycle progression as null mutants of an asynchronous population accumulated in G2 phase relative to wild type cells [28].

It is thus well established that SIN3 regulatory function is important for cell proliferation and, in *Drosophila* and mouse models, is required for early developmental progression. Little is understood, however, regarding the role of SIN3 during adulthood, after the majority of developmental cell proliferation has occurred. In this study, we set out to address the role of SIN3 during the adult stage of the *Drosophila* life cycle. Using a conditional knockdown system, we allowed the flies to develop to adulthood and then initiated RNAi induced *Sin3A* knockdown to reduce expression levels. Utilizing this system we find that reduced SIN3 levels affect the locomotor ability of the flies. Additionally, consistent with cultured cell studies, SIN3 is important for tolerance to stress. In line with the observed oxidative stress sensitive phenotype, we find that knock down of *Sin3A* results in a shortened adult lifespan. This result was somewhat surprising given previous studies indicating that inhibition of HDAC activity resulted in an increase in longevity [29]. We did not observe gene expression changes to strongly support a role for SIN3 as a key regulator of longevity gene activity. Instead, genes important for an oxidative stress response were altered upon reduction of SIN3. Taken together, the data suggest that SIN3 regulates signaling processes and a stress response critical for maintenance of a healthy adulthood.

## RESULTS

### Generation and initial characterization of conditional knockdown flies

Because SIN3 is required for embryonic viability, we previously developed a conditional RNAi knockdown transgenic fly to target *Sin3A* degradation [17]. RNAi induction through the GAL4-UAS system was used to reduce SIN3 levels [30, 31]. To examine the effect of *Sin3A* knockdown in adult flies, we used the mifepristone (RU486) inducible GAL4 Gene-Switch [32]. The *GAL4* transgene is driven by the ubiquitous β-tubulin promoter. The GAL4 activator is only active in the presence of RU486. Flies were generated that carried both the Gene-Switch GAL4 and a *UAS-SIN3^RNAi^* transgene for expression of *Sin3A* double stranded RNA (dsRNA). To demonstrate that the dsRNA produced by induction of the transgene is specific for *Sin3A* and ensure that observed phenotypes are not due to an off target effect, we used two constructs to target different exons within the *Sin3A* transcript [18, 33]. Flies with these constructs are referred to as -I or -II. Progeny of the cross between the UAS-SIN3^RNAi^ lines and the GAL4 Gene-Switch line are referred to as SIN3 KD I and SIN3 KD II. For each experiment, flies were reared on food with RU486, referred to as knockdown, or without, referred to as control. It was recently reported that under some growth conditions, induction of a sequence-independent RNAi response could affect lifespan [34]. Therefore, as an additional control, we used the GAL4 Gene-Switch to induce RNAi of green fluorescent protein (GFP), which is not expressed in *Drosophila*. In this way, the RNAi machinery is activated by expression of GFP dsRNA but there is no mRNA target present. Progeny of the cross between the UAS-GFP^RNAi^ line and the Gene-Switch GAL4 line are referred to as GFP RNAi. Any differences in the progeny with or without RNAi induction were noted. We refer to this comparison as the RNAi control.

**Figure 1:**
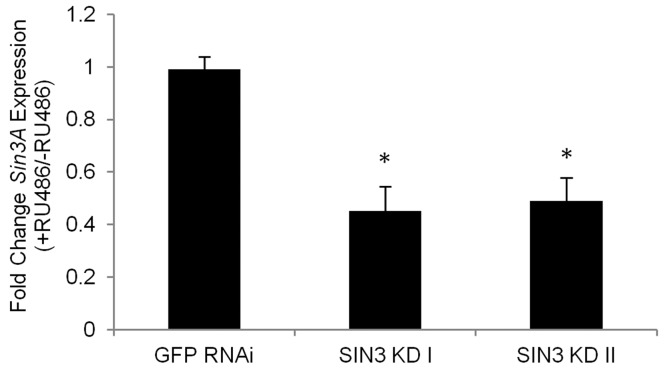
The Gene-Switch GAL4 driver can be used to effectively knock down expression of *Sin3A* in adult *Drosophila* RT-qPCR analysis was performed using tissue isolated from the indicated fly lines without RU486 (control) or with RU486 (RNAi knockdown). cDNA prepared from 25 day-old flies was used as a template in PCR with primer pairs for *Sin3A*. Numbers I and II refer to two distinct lines designed to express dsRNA to target different regions of the *Sin3A* transcript. Relative levels of gene expression are indicated. Error bars represent standard error of the mean. * p<0.01.

To test the efficiency of conditional knock down of *Sin3A* using the Gene-Switch system, total RNA was extracted from 25 day-old flies and RNA levels for *Sin3A* were measured using quantitative PCR following reverse transcription. RNA levels in the knockdown flies were reduced to less than 49% of control flies using either targeting construct (Fig. 1).

### Locomotor activity is affected by alteration of SIN3 levels

**Figure 2:**
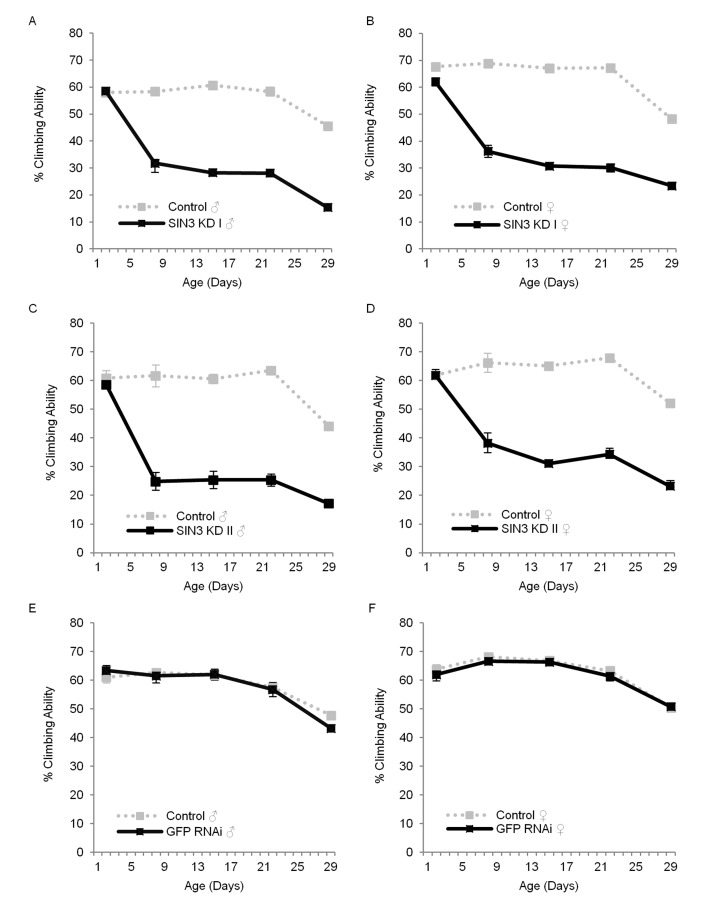
*Sin3A* knockdown (KD) affects locomotor function Climbing ability of control (-RU486), SIN3 KD or GFP RNAi (+RU486) flies is indicated. The average percentage of flies that crossed the 30 cm mark on a 30 ml graduated cylinder in a 30 second time period, measured on indicated days is plotted. Error bars represent standard error of the mean. **A** and **B**. SIN3 KD I, **C** and **D**. SIN3 KD II, **E** and **F**. GFP RNAi serves as an additional control. p<0.001 for the comparison of control and SIN3 KD for each data set starting at day eight.

When culturing the *Sin3A* knockdown flies, we noticed that as they aged, they were increasingly lethargic and had reduced ability to fly when compared to their wild type counterparts. Additionally, they frequently fell off the walls of the culture vial and easily stuck to the food. Because healthy muscle tissue utilizes ATP and requires functional mitochondria, which are regulated in part by SIN3 [12], we asked if SIN3 is important for normal locomotor function. We used a standard climbing assay [35, 36] to measure the ability of flies to climb or fly up a graduated cylinder, examining both sexes as they aged. At two days post-eclosion, there was little or no difference in climbing ability (Fig. 2), but as early as eight days of age, both male and female *Sin3A* knockdown flies showed a dramatic decrease in their ability to climb (Fig. 2A-D), which continued for the duration of the assay. To rule out a possible effect of the RNAi response, we measured the climbing ability of GFP RNAi flies with and without RU486. We did not observe any difference (Fig. 2E, F). The exacerbation of the decrease in locomotion over time is consistent with many studies showing that climbing ability decreases as a function of age [36-38]. These results demonstrate that SIN3 is critical for wild type locomotor activity throughout much of the lifespan of the adult fly.

### SIN3 is critical for tolerance to acute oxidative stress

**Figure 3:**
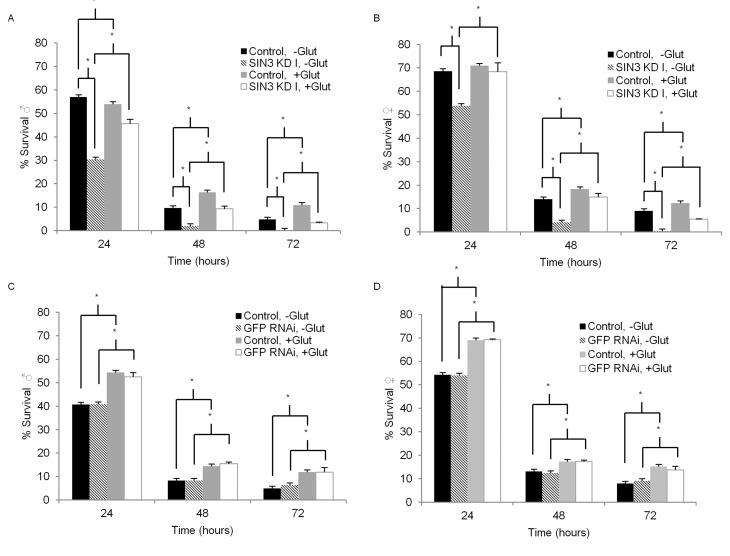
Enhanced *Sin3A* knockdown (KD) paraquat sensitivity is partially suppressed by glutathione Survival of control (-RU486), SIN3 KD or GFP RNAi (+RU486) flies on 5 mM paraquat is indicated. For glutathione treatment, flies were placed on media containing 0.22mM glutathione for seven days prior to paraquat treament. Error bars represent standard error of the mean. **A** and **B**. SIN3 KD. **C** and **D**. GFP RNAi serves as an additional control. * p<0.01.

Mitochondrial function is important not only for ATP generation, but also to mount an effective stress response [39]. Previous studies have demonstrated that SIN3 is important for mitochondrial biogenesis and activity, including the removal of reactive oxygen species (ROS) [9, 12]. To examine the role of SIN3 in tolerance to oxidative stress, we used paraquat to induce acute oxidative stress in adult flies with wild type or reduced SIN3 levels. *In vivo*, following a NADPH-dependent reduction, paraquat yields a stable radical that reacts with oxygen to make the superoxide anion, a ROS [40]. Oxidative stress ensues when ROS accumulate, causing damage to proteins, lipids, and nucleic acids [41]. Paraquat is used extensively in *Drosophila* to examine the effects of oxidative stress [42]. Survival of male or female flies incubated with paraquat was measured each day for three or four days. Males with ubiquitous knockdown of *Sin3A* using either exon targeting sequence (SIN3 KD I or SIN3 KD II) exhibited heightened sensitivity to paraquat as compared to control flies with no RNAi activation (Fig. 3A, compare black and hatched bars, Supplemental Fig. 1). Similarly, *Sin3A* knockdown females showed increased sensitivity (Fig. 3B, Supplemental Fig. 1).

Male and female flies with GFP RNAi exhibited no increase in paraquat sensitivity relative to matched controls (Fig. 3C, D). Survival of males and females on control filters containing 5% sucrose (0 mM paraquat) with ubiquitous *Sin3A* knockdown or GFP RNAi showed no difference when compared to the control flies without activation of the Gene-Switch (Supplemental Fig. 1 and 2). Taken together, these data show that ubiquitous reduction of SIN3 levels results in heightened sensitivity to paraquat-induced oxidative stress.

One way in which cells respond to an increase in the production of ROS is through the oxidation of glutathione (c-glutamyl-cysteinyl-glycine, GSH), as it donates an electron to these unstable species, resulting in the disulfide GSSG [43, 44]. Dietary glutathione supplementation has been shown to counteract the toxicity of paraquat, resulting in the increased survival of flies in paraquat sensitivity assays [45]. We therefore asked if glutathione supplementation could affect the tolerance of *Sin3A* knockdown flies to paraquat treatment. For these experiments, after eclosion, flies were placed on standard molasses food supplemented with 0.22 mM glutathione for one week. Next, similar to experiments described above, survival of male or female flies incubated with paraquat but in this case, supplemented with glutathione, was measured each day for three days. Consistent with previous reports [45], glutathione supplementation increased survival of nearly all flies treated with paraquat (Fig. 3A-D and Table 1). Interestingly, the effect was much more pronounced in flies in which SIN3 levels were reduced. In males with ubiquitous knockdown of *Sin3A*, glutathione supplementation ameliorated the strong paraquat sensitivity seen in the un-supplemented flies (Fig. 3A, compare light grey and white bars and Table 1). Similarly, in females with ubiquitous knockdown of *Sin3A*, glutathione supplementation increased the tolerance to paraquat over and above levels observed in the wild type counterparts (Fig. 3B, D, Table 1). For both sexes, the difference in paraquat sensitivity upon glutathione supplementation increased with increasing time of exposure (Table 1). In comparison, flies with wild type levels of SIN3 showed some resistance to paraquat toxicity with glutathione supplementation, but their survival rates did not increase dramatically and were relatively unchanged over the course of the experiment. GFP RNAi control flies also showed some level of increased resistance to paraquat toxicity upon glutathione supplementation, consistent with previously published reports [45]. Again, the increase in paraquat tolerance was much less than that observed in the *Sin3A* knockdown flies (Table 1). Glutathione supplementation had no effect on survival for the control conditions of 5% sucrose and no paraquat treatment (Supplemental Fig. 2). Results from this set of experiments indicate that SIN3 is important for responding to oxidative stress, possibly through regulation of cellular glutathione.

**Table 1:** Glutathione supplementation increases the tolerance of SIN3 KD flies to paraquat

Genotype	Sex	Relative Survival +Glutathione/-Glutathione					
		24		48		72	
		Control	KD	Control	KD	Control	KD
SIN3 I	♂	0.95	1.5	1.69	4.67	2.3	*
	♀	1.03	1.27	1.31	3.62	1.37	16.33
GFP	♂	1.34	1.28	1.74	1.86	2.37	1.88
	♀	1.27	1.28	1.33	1.4	1.92	1.53

Control, flies without RU486; KD, flies with RU486; 24, 48, 72 = time in hr.

* All flies without glutathione supplementation were dead. Ratios for each time point were calculated by dividing the number of flies surviving with glutathione supplementation by the number of flies surviving with no glutathione supplementation.

### Alterations in SIN3 expression modulates adult lifespan

**Figure 4:**
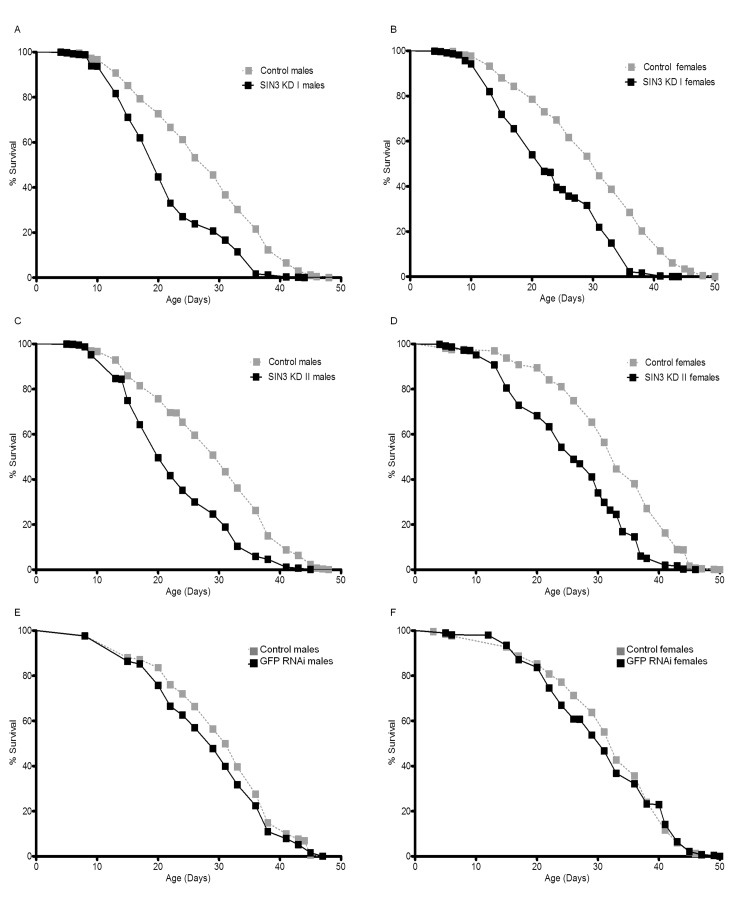
Ubiquitous *Sin3A* knockdown (KD) causes decreased survival Mean survival of control (-RU486), SIN3 KD or GFP RNAi (+RU486) flies is indicated. **A** and **B**. SIN3 KD I (p<0.0001 using the logrank (Mantel-Cox) test), **C** and **D**. SIN3 KD II (p<0.0001), **E** and **F**. GFP RNAi serves as an additional control.

Sensitivity to ROS has been linked to effects on longevity since the mid-1950s when the “free-radical theory” of aging was postulated [46, 47]. Additionally, susceptibility to paraquat toxicity has been correlated with reduced lifespan [42, 48]. As reduction in SIN3 levels has previously been linked to oxidative stress [9] and the adult *Sin3A* knockdown flies are sensitive to paraquat (Fig. 3, Supplemental Fig. 1), we investigated whether reduction of SIN3 affected longevity. To determine if SIN3 plays a role in lifespan, males or females were reared on food with or without RU486 for approximately 50 days. Males and females with ubiquitous knock down of *Sin3A* using either exon targeting dsRNA (SIN3 KD I or SIN3 KD II) exhibited decreased lifespan as compared to control flies with no RNAi activation (Fig. 4A-D). GFP RNAi flies did show a slight decrease in lifespan as compared to matched controls (Fig. 4E-F), but the difference in median and maximum lifespan in comparison to their control counterparts was not statistically significant (Table 2). The median survival (LT50) for the SIN3 KD I and SIN3 KD II flies was much lower than their control counterparts (Table 2). The difference in maximum lifespan (LT90) for the SIN3 KD I and SIN3 KD II flies compared to their control counterparts was even greater than the difference for the LT50 (Table 2).

**Table 2:** Ubiquitous reduction of SIN3 levels causes a decrease in both median survival and maximum lifespan

Genotype	Sex	LT50 in days ± SD		p value	Relative change in LT50	LT90 in days ± SD		p value	Relative change in LT90
		Control	KD			Control	KD		
SIN3 I	♂	29±1.2	22±0.0	0.02	0.71	37 ± 0.6	28 ± 1.5	0.02	0.74
	♀	31±1.7	22±0.0	0.02	0.69	44 ± 1.5	27 ± 1.7	0.02	0.62
SIN3 II	♂	31±1.2	20±3.1	0.00	0.79	42 ± 1.5	26 ± 0.6	0.05	0.62
	♀	33±0.0	26±1.2	0.02	0.65	42 ± 1.0	30 ± 0.6	0.00	0.71
GFP	♂	31±0.6	29±1.2	0.58	0.94	41 ± 0.0	39 ± 1.2	0.29	0.96
	♀	33±1.2	31±3.6	0.12	0.94	41 ± 1.2	42 ± 2.1	0.21	1.01

LT50, median survival; LT90, maximum survival; Control, flies without RU486; KD, flies with RU486; SD, standard deviation, calculated for the average of three trials.

While these results demonstrating that a reduction of SIN3 leads to reduced longevity are consistent with the locomotor and paraquat sensitivity assays, they are somewhat surprising in light of previously published work indicating that inhibition of HDAC complexes with chemical inhibitors or haplo-insufficiency of *Rpd3* leads to increased longevity [49-53]. Possibilities for these differing results will be addressed below. Data obtained from this study indicate that *Drosophila* *Sin3A* is important for normal lifespan and that reduced expression of this essential gene leads to shorter life.

### Effect of reduced SIN3 levels on expression of genes involved in aging and stress tolerance

**Figure 5:**
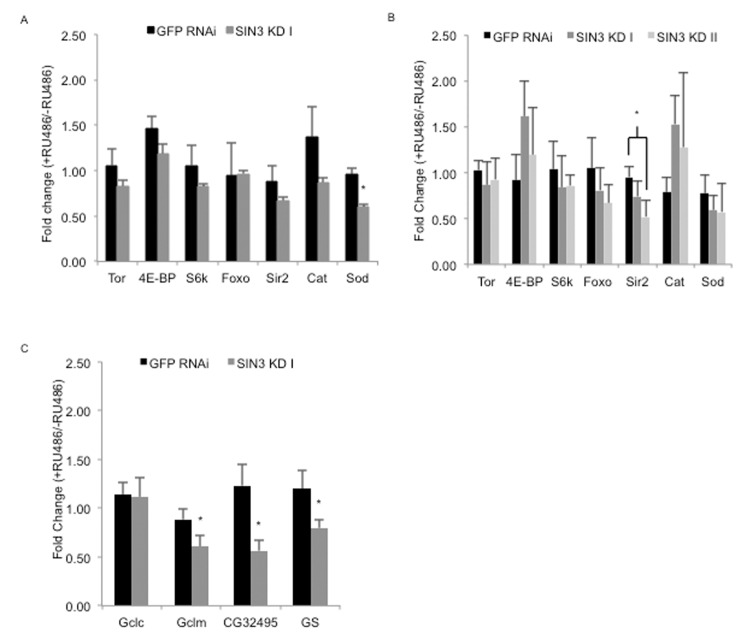
Effects of ubiquitous knock down of *Sin3A* on expression of genes known to impact longevity and glutathione synthesis RT-qPCR performed using cDNA prepared from RNA isolated from whole flies from control (-RU486), SIN3 KD or GFP RNAi (+RU486). Fold changes in gene expression for GFP RNAi, SIN3 KD I and II are shown for predicted longevity relevant genes in seven day- (**A**) or 20 day-old flies (**B**) and genes important for the de novo synthesis of glutathione in seven day-old flies (**C**). Error bars represent standard error of the mean. * p<0.05.

There is a long-standing interest in the potential link between ROS and aging [47]. Genes involved in removal of ROS have previously been determined to be targets of SIN3 [9, 12]. To test if reduction of SIN3 leads to altered expression of genes associated with longevity and other phenotypes observed in the *Sin3A* knockdown flies, we monitored mRNA levels of a number of candidates. We isolated mRNA from *Sin3A* and GFP RNAi and control flies at day seven, the age of the flies tested in the paraquat assay, and at day 20, a time point in which we observed a strong difference in survival in the longevity study (Figs. 3, 4). This mRNA was converted to cDNA and used in a quantitative-PCR assay (Fig. 5). Multiple components of the TOR signaling pathway have been found to be involved in regulation of the aging process [54]. We analyzed the gene expression level of three components in the pathway including *tor, Thor* (also known as *4E-BP*) and *RPS6-p70-protein kinase (S6k)*. Of these, only *4E-BP* exhibited a trend toward up-regulation following a reduction in SIN3 levels. A second pathway implicated in aging is through Forkhead box, sub-group O (FOXO), a transcription factor target of insulin signaling [55]. *Foxo* expression is mildly down-regulated in the *Sin3A* knockdown flies, only in the 20 day-old flies. This finding is consistent with the observation that overexpression of *foxo* leads to increased longevity [55, 56]. Although the potential role of the class III HDAC *Sir2* in aging has recently come into question, recent work has shown that dSir2 in the adult fat body regulates longevity in a diet-dependent manner [57, 58]. In the *Sin3A* knockdown flies, the expression of *Sir2* is mildly decreased, which is consistent with previous findings indicating that *Sir2* overexpression leads to increased longevity [59]. Catalase is an important enzyme for removal of ROS [41]. Catalase expression is up-regulated following *Sin3A* knockdown in *Drosophila* S2 cells [9]. Consistent with the effects in cultured S2 cells, *Cat* expression was mildly increased in 20 day-old flies compared to the control. Altered expression of *Superoxide dismutase (Sod)* has been found to affect *Drosophila* longevity, with overexpression extending lifespan while reduced expression shortens lifespan under conditions of a high sugar-low protein diet [60, 61]. *Sod* expression showed a moderate and statistically significant decrease in the seven day-old flies and a mild decrease relative to control in 20 day-old flies. Taken together, while there is a trend toward a decrease in expression of “longevity” genes in the *Sin3A* knockdown flies, the small differences in expression do not strongly support the idea that SIN3 is a key regulator of aging genes.

Based on the findings detailed above, we hypothesized that the decreased lifespan is likely due to cumulative effects of altered expression of genes involved in numerous developmental and stress response pathways, possibly including the de novo synthesis of the antioxidant glutathione. Synthesis of glutathione, from cysteine, glutamate and glycine, is catalyzed by the enzymes glutamate–cysteine ligase and glutathione synthetase. Given that glutathione supplementation rescued the observed sensitivity of *Sin3A* knockdown flies to paraquat, we asked whether enzymes responsible for glutathione synthesis were regulated by SIN3. We measured expression of the four genes encoding these enzymes, *Glutamate-cysteine ligase catalytic subunit (Gclc), Glutamate-cysteine ligase modifier subunit (Gclm), CG32495* and *Glutathione synthetase* (*GS)*, by a quantitative-RT-PCR assay. For this experiment, we used RNA isolated from seven-day old flies, the age of the flies tested in the paraquat assay. *Gclm, CG32495* and *GS* all showed a significant decrease in expression in *Sin3A* knockdown flies relative to controls (Fig. 5C). These results strongly suggest that SIN3 is important for normal longevity by modulating the stress resistance response in the adult fly, possibly through control of genes involved in the de novo synthesis of glutathione.

## DISCUSSION

In this study we have addressed the role of the essential gene *Sin3A* in adult *Drosophila*. The data indicate that SIN3 is critical for locomotor function, resistance to oxidative stress and longevity. Previous studies have demonstrated a role for SIN3 during development [15-17]. Results presented here show that, in addition to participating in development and cell proliferation, SIN3 performs critical functions in differentiated tissue. The observed adult phenotypes indicate that SIN3 is important throughout the lifespan of *Drosophila*. The findings support a model in which the SIN3 corepressor is a key player in the stress response required to counter-act cellular assaults encountered during adulthood.

One of the tests we performed on the *Sin3A* knockdown adult flies was a paraquat sensitivity assay. Paraquat induces ROS that in turn promote oxidative stress that eventually results in mitochondrial dysfunction [40, 62]. We and others have found that reduction of SIN3 leads to changes in expression of genes important for mitochondrial function in *Drosophila* and mouse [8, 9]. Additionally, RNAi knock down of *Sin3A* in *Drosophila* cells resulted in aberrant mitochondrial physiology, with cells having altered cellular respiration and ATP production [12]. Yeast cells harboring a null mutation in ySin3 have low levels of ATP and are unable to grow when cultured under conditions that require respiration [12]. Consistent with the observations obtained in the *Drosophila* cell culture system, we observed that adult flies with *Sin3A* knockdown are highly sensitive to paraquat, and thus to oxidative stress, relative to their wild type counterparts (Fig. 3, Supplemental Fig. 1). Importantly, this sensitivity can be rescued by dietary supplementation of the antioxidant glutathione. Gene expression analysis of four genes required for glutathione synthesis indicates *Sin3A* is important for expression of three of the four genes (Fig. 5C). The effect of reduced SIN3 on expression of genes involved in glutathione synthesis may contribute to the reduced longevity phenotype. Overexpression of *Gclc* or *Gclm* leads to extended longevity [43, 63]. Additionally, reduction of *Gclc* in specific tissues resulted in reduced lifespan [63]. While effects on *Gclc* expression were not observed in the *Sin3A* knockdown adults, the other components of the de novo glutathione synthesis pathway exhibited reduced expression (Fig. 5C). We hypothesize that SIN3 indirectly affects the expression of the glutathione synthesis genes for two reasons. Firstly, the expression of these genes is reduced in the *Sin3A* knockdown flies rather than upregulated, as might be expected as SIN3 is more typically associated with transcription repression [64]. Secondly, for genes in which the data are available on modENCODE, SIN3 does not exhibit strong binding to any of the genomic regions that encode the glutathione synthesis genes [65].

In addition to the paraquat sensitivity, the reduced locomotor activity suggests that mitochondrial function may be disrupted in the *Sin3A* knockdown adults. Climbing ability requires active muscle tissue, which utilizes the energy of ATP, the majority of which is produced in mitochondria. Taken together, the paraquat sensitivity and reduced locomotion are strongly supportive of the idea that SIN3 is a critical regulator of cellular energy production and an active stress response, possibly through maintenance of functional mitochondria and regulation of enzymes required for oxidative stress management.

There is a large body of evidence linking an increase in ROS and oxidative stress with the aging process [66, 67]. Mitochondria are a major source of ROS in the cell and mitochondrial function has been found to decline in aging organisms [68]. Given the changes in expression of genes encoding mitochondrial proteins observed in *Sin3A* knockdown cultured cells, it is perhaps not surprising that the *Sin3A* knockdown flies have reduced longevity compared to wild type controls. Interestingly, *Sin3A* was recently isolated as a positive hit in an overexpression screen for genes regulating *Drosophila* longevity [69]. The insertion of an engineered P-element promoting expression of the neighboring target by GAL4 activation resulted in an approximately three-fold increase in *Sin3A* expression. This led to a significant increase in mean lifespan relative to the controls. Thus, the amount of SIN3 is critical for normal longevity, either too much or too little, can affect the process. We note that the observed maximum lifespan of the flies in this investigation is shorter than that of the above study as well as many others. For our experiments, flies were reared at 27°C, rather than a standard 25°C, to promote the activity of the GAL4 activator [30]. It has been reported that longevity is dependent on temperature [70]. The short lifespan of the control flies observed in our study is thus possibly due the growth conditions.

Additional factors with biochemical and/or genetic links to SIN3 activity have also been found to regulate the length of the adult lifespan. The histone demethylase Little imaginal discs (LID) was found to be a component of a SIN3 complex [71, 72]. Similar to what we have observed in the *Sin3A* knockdown flies, male flies expressing a demethylase mutant of LID are sensitive to paraquat treatment and have reduced longevity as compared to wild type controls [73]. SIN3 has also been found to interact with the steroid hormone ecdysone receptor (EcR) through the corepressor SMRTER and localizes to EcR targets on polytene chromosomes [74, 75]. Strong RNAi knockdown of *EcR* leads to a decrease in longevity [76]. Thus, effects in adulthood may be mediated in part through action of EcR-regulated genes. Interestingly, a moderate rather than large reduction in EcR led to enhanced longevity in males and reduced longevity in females, serving as an additional example that longevity phenotypes can vary with alterations in the dose of the effectors [76].

SIN3 serves as a scaffold for assembly of a transcriptional corepressor complex with HDAC activity due to the presence of the RPD3 enzyme [64]. Rather than reducing lifespan, haploinsufficiency of *Rpd3* results in extended longevity [52]. Additionally, alterations in mean lifespan have been observed in flies cultured on various HDAC inhibitors including Trichostatin A (TSA), 4-phenobyturate, suberoylanilide hydroxamic acid (SAHA) and sodium butyrate (NaBu) [49-51, 53]. The amount of drug administered in these studies is important, as high concentrations are lethal. Interestingly, different effects on lifespan are noted dependent on dose and time of administration of drug. In the most recent study, feeding on NaBu during only the initial stages (days 1-21) or throughout adulthood led to a decrease in longevity, while feeding only after 21 days led to extended longevity [49]. SAHA had the same time specificity as did NaBu in these studies, suggesting that this phenomenon may be a characteristic of class I HDAC inhibitors [49]. Collectively, these studies implicate regulation of histone acetylation levels as a critical process throughout the lifetime of an organism. Further, they suggest that changes to acetylation patterns may alter gene expression programs required for longevity.

SIN3 is a widely expressed corepressor that regulates histone modifying activity [3]. Research in multiple model systems including *Drosophila* and mouse indicate that SIN3 regulates processes critical for cell proliferation and development [8, 13, 14, 16-18]. Here we show that SIN3 is important not only during the early developmental stages, but also in *Drosophila* adulthood. SIN3 is considered a global transcriptional regulator [64]. We predict that the altered longevity and stress response found in adult flies having reduced levels of SIN3 is due to the change in expression of a set of coordinately regulated genes rather than due to the activity of a single SIN3 regulated gene. Expression profiling in cultured cells isolated from embryos indicates that genes encoding mitochondrial proteins and those involved in cellular bioenergetics are subject to SIN3 regulation [9]. Additionally, in this work, we determined that genes involved in synthesis of the antioxidant glutathione are altered when SIN3 levels are reduced. Future studies will determine if the phenotypes associated with SIN3 reduction in adulthood are due to defects in energy production and/or misregulation of genes required to initiate and maintain an adequate stress response.

## METHODS

### Fly stocks

*Drosophila melanogaster* stocks were maintained and crosses were performed according to standard laboratory procedures. The RU486-inducible Gene-Switch GAL4 driver, *yw; +, tubulin-Gene-Switch*, was a gift from S. Pletcher (University of Michigan, Ann Arbor, MI). Generation of the transgenic flies carrying the *UAS-SIN3^RNAi-I^* and *UAS-SIN3^RNAi-II^* transgenes are described previously [17, 18]. UAS-GFP^RNAi^ (#9331) was obtained from the Bloomington Drosophila Stock Center.

### Reverse transcription PCR assays

Total RNA was extracted from females using Trizol (Invitrogen) and purified using the RNeasy mini kit (Qiagen). cDNA was generated from total RNA using the ImProm-II Reverse Transcription System (Promega) with random hexamers. The cDNA was used as template in a quantitative real-time PCR (qPCR) assay. The analysis was performed using ABsolute Blue SYBR Green ROX master mix (Fisher Scientific) and carried out in a Stratagene Mx3005P real-time thermocycler. Taf1 and Pgk primers were used to normalize the RNA levels. To examine gene expression changes that result from ubiquitous reduction of SIN3 levels, candidate genes involved in Tor signaling and/or longevity (*tor*, *4E-BP*, *S6k*, *Sod*, *Cat*, *Foxo*, *Sir2*) or in the paraquat-induced stress response (*Gclc, Gclm, CG32495 and GS*) were chosen. The mRNA levels (of seven or 20 day-old flies for Tor signaling and/or longevity genes or seven day-old flies for the glutathione synthesis genes) were quantified using real time PCR with specific primers for each gene (Supplemental Table 1). The gene expression changes are represented as the mean (± SEM) of the fold changes observed in the fly lines (GFP RNAi, SIN3 KD I and SIN3 KD II) with and without RU486. The fold differences were calculated by relative comparison of each fly line with RU486 to its respective counterpart without RU486, [GFP RNAi (+RU486)/GFP (-RU486), SIN3 KD I (+RU486)/SIN3 KD I (-RU486) and SIN3 KD II (+RU486)/SIN3 KD II (-RU486)]. This experiment utilized a minimum of three sets of RNA for each fly line.

### Climbing assay

A climbing assay was performed as described previously [35, 36], with minor modifications. Following anesthesia under light CO_2_, ten males or females were placed in a 30 cm × 1.5 cm graduated plastic cylinder and allowed to equilibrate for 30 min. The flies were gently tapped to the bottom with two to three taps and allowed to climb or fly. The number of flies that crossed the 30 cm mark on the cylinder in a 30 second time period was recorded. This procedure was repeated with the same group of ten flies for ten trials for each sex, allowing one min rest intervals between trials. This experiment was repeated once each week from day two post-eclosion to day 29, for three biological replicates, for a total of 300 flies per genotype and sex.

### Paraquat stress resistance and glutathione supplementation

Seven day-old flies were tested for their resistance to paraquat (1 1,10-dimethyl-4,40-bipyridinium dichloride) (Sigma Aldrich). 25 male or female flies were anesthetized under light CO_2_ and then transferred to vials containing five 2.3 cm, grade 3 filter paper circles (Whatman) saturated with 200 μl of either 5% sucrose solution or 5 mM paraquat in 5% sucrose solution. Flies were kept in the dark at all times, except for scoring at 24, 48 and 72 hr. Five vials of each sex were scored. This experiment was repeated three times, for a total of 375 flies per genotype and sex. Glutathione supplementation experiments were conducted as above, with the exception that 0.22 mM glutathione was added to the media immediately after eclosion and to the filter paper circles.

### Longevity

Flies were collected for a 24 hour period after eclosion and reared at 27°C, to promote GAL4 activity [30], for 48 hours on standard sucrose yeast media. Flies were sorted by sex under light CO_2_ anesthesia into vials with 200 mM final concentration of RU486 (dissolved in 5% ethanol) or 5% ethanol for lifespan analysis. Ten vials of 25 flies of each sex were transferred to fresh food three times each week, and dead flies were removed and recorded. This experiment was repeated three times, for a total of 750 flies per genotype and sex.

### Statistics

An unpaired Student's *t*-test was used to determine significance for gene expression, paraquat sensitivity and climbing ability assays (GraphPad). Life-span curves and statistical analysis was performed using Prism4 software (GraphPad).

## SUPPLEMENTAL DATA


