# Supplementary material for: SIN3 is critical for stress resistance and modulates adult lifespan
Source: Aging (Albany NY). 2014 Aug 7;6(8):645–60. doi: 10.18632/aging.100684 (PMC4169859; doi:10.18632/aging.100684)
Supplement: Supplementary file 1 [file aging-06-645-s001.pdf]

SUPPLEMENTAL DATA

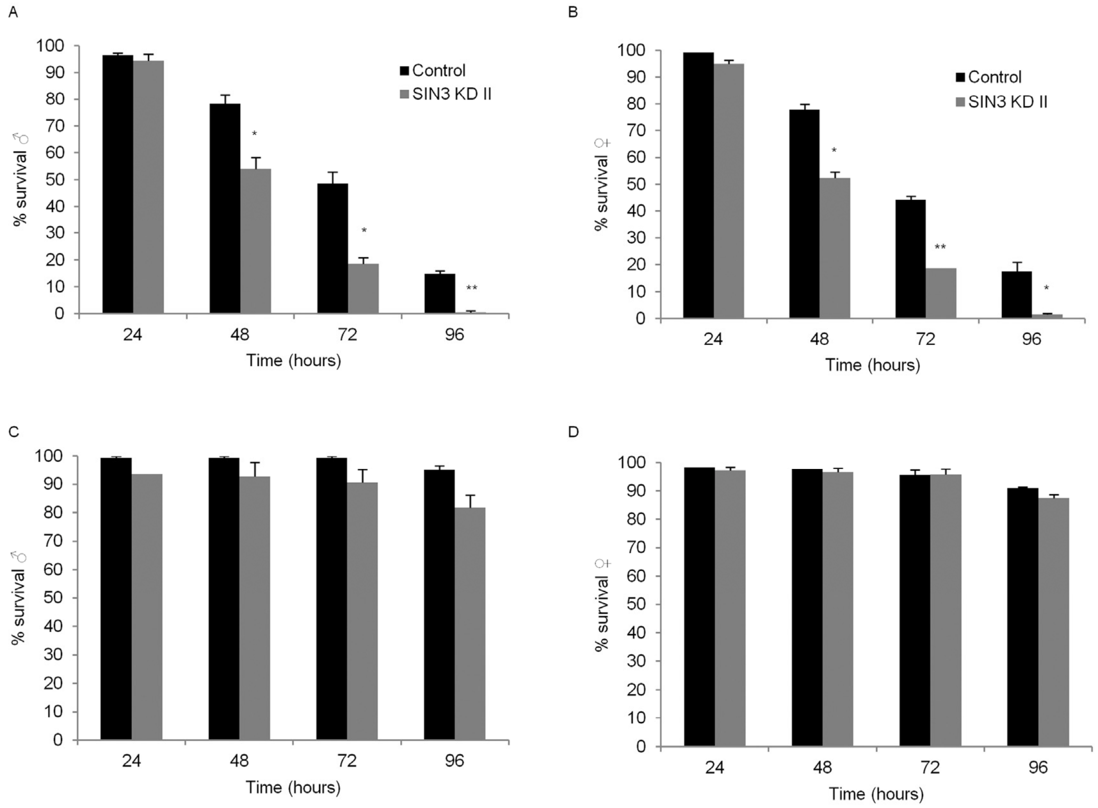

Supplemental Figure 1. (**A, B**) Ubiquitous *Sin3A* knockdown (KD II) results in greater sensitivity to paraquat. Survival of control (-RU486) or SIN3 KD II (+RU486) flies on 3 mM paraquat is indicated. (**C, D**) *Sin3A* knockdown (SIN3 KD II) does not significantly affect survival on 5% sucrose (0 mM paraquat). Error bars standard error of the mean. * p<0.01, ** p<0.001.

Supplemental Table 1. Primer pairs used in this study

Gene	Forward primer sequence 5' -> 3'	Reverse primer sequence 5' -> 3'
Sin3A	TTGTCTCCAATGCTGTTCGC	GGTTGGCGAATCCTGCGCTC
Taf1	GTGGAGGAGCCAAGGGAGCC	TCCCGCTCCTTGTGCGAATG
Pgk	CCCCCCGGTGTCTTTGAG	GCCGTCCATGATGGACTTG
Tor	ACTGGAGACTGTTGGATGTGGAC	GCTGTAGAGTCGGATCGTAGG
S6k	CGCTGCTGTTCTGTTCTGTACTG	GGCGAGTGCGATTCTGTTTGG
4E-BP	GCTAAGATGTCCGCTTCACC	CCCGCTCGTAGATAAGTTTGG
Foxo	AGCAGCAGCAACAGCAACAG	GCCAGCGGTATATTGATGTCCAG
Sir2	CAATAAGGAGCAGAGCGTGGATG	TTCAGCGAGGAGCCGATCAC
Cat	GCCTTGTCCTCCTGCTGTCC	GGCTGGCGTTGCTCAAATGG
Sod	AACATCACCGACTCCAAGATTACG	CAATAACGCCGCACCCGATG
Gclc	CTCACCACGGAATCCTGC	GCTAATCACCACGGCCAC
Gclm	CATTCCGTCCATTCTCCG	GCGTGGTGGTTGAAAAGG
GS	GGGCCAGTCCAAGGAGTT	TCGTAGCCAGCTCGGAAG
CG32495	GCGCAAGGAGTTCGAAAA	AGATTGGCCGTGAACTCG

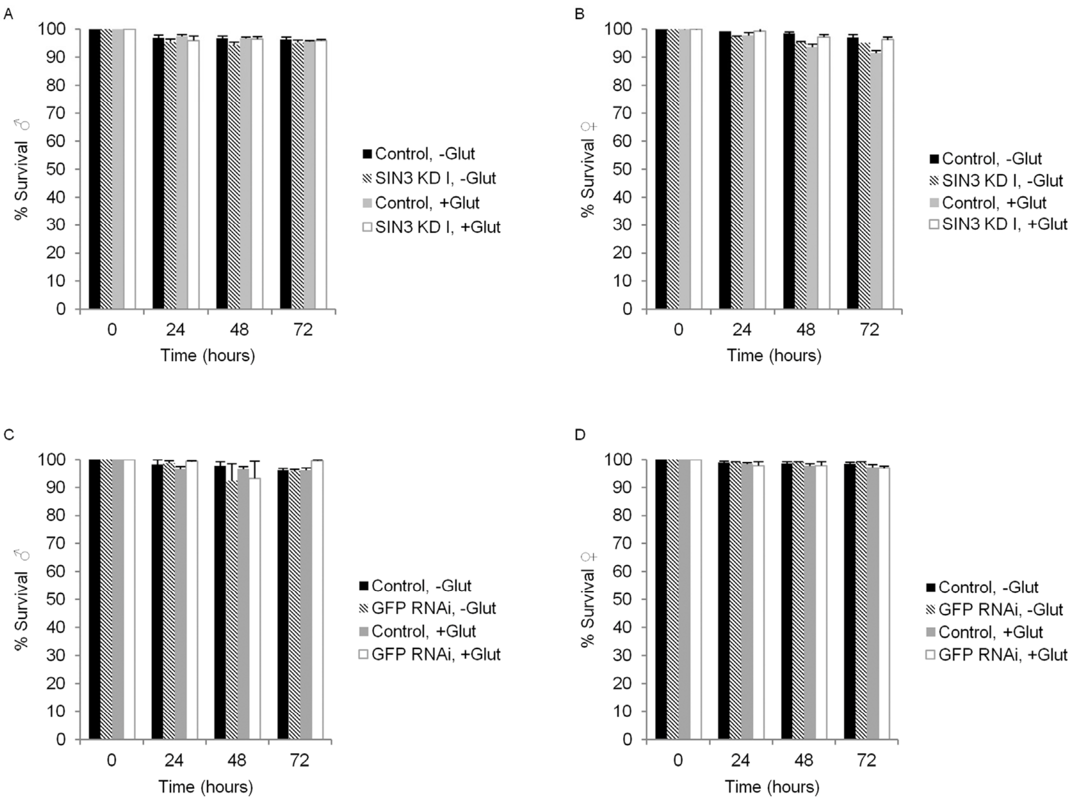

Supplemental Figure 2. *Sin3A* knockdown (KD I) does not affect survival on 5% sucrose with or without glutathione supplementation. Survival of control (-RU486), SIN3 KD I or GFP RNAi (+RU486) flies with the addition of 0.22 Mm glutathione (+Glut) or no glutathione (-Glut) on 5% sucrose (0 mM paraquat) is indicated. Error bars standard error of the mean. **A** and **B** SIN3 KD I; **C** and **D** GFP RNAi serves as additional control.
